# Triadic Interactions in MIECHV: Relations to Home Visit Quality

**DOI:** 10.1007/s10995-018-2534-x

**Published:** 2018-06-12

**Authors:** Carla A. Peterson, Kere Hughes-Belding, Neil Rowe, Liuran Fan, Melissa Walter, Leslie Dooley, Wen Wang, Chloe Steffensmeier

**Affiliations:** 1 Human Development and Family Studies, Iowa State University, 4380 Palmer, Ames, IA 50011 USA; 2 1331 Palmer Building, 2222 Osborn Drive, Ames, IA 50011-1084 USA

**Keywords:** Home visiting quality, Triadic interactions, Evaluation

## Abstract

*Objectives* This study was conducted to look inside home visits to examine active intervention ingredients used and their relations with ratings of home visit quality. In particular, triadic interactions that engage the home visitor, parent, and child together and provide a context for home visitors to facilitate parent-child interactions by observing, modeling and coaching behaviors that promote optimal child development were examined. *Methods* Observations were conducted to describe intervention activities (with the HVOF-R) and rate quality of home visit practices and engagement (with the HOVRS A+). *Results* Analyses revealed the majority of home visit time (71%) was spent in home visitor-parent interactions with only a small proportion of home visit time (17%) spent in triadic interactions and an even smaller proportion of time (2%) during which home visitors actively coached parent-child interactions. Amount of time spent in triadic interactions was related positively to quality ratings of home visit practices and engagement. Moreover, time spent coaching parent-child interactions uniquely predicted home visit quality after accounting for visit length and home visitor time spent observing and modeling. *Conclusions for Practice* Increasing the percentage of home visitors engage the parent and child in triadic interaction should be a focus for home visiting programs. Home visitors will likely need professional development and supervisory support to enhance their skills in coaching parent-child interactions during triadic interactions.

## Significance

*What is already known on this topic?* Home visiting programs that support parenting practices for families facing risks have been linked to gains in positive parenting practices and reductions in child maltreatment. In addition, participation in evidence informed home visiting programs improves children’s developmental outcomes.

*What does this study add?* This manuscript includes a detailed examination of the relations among specific interactions among home visitors, parents and children and home visit quality. Results of this study provide a significant contribution to professional development efforts by elucidating specific interactions differentially associated with home visit quality.

## Introduction

Home visiting programs are designed to promote child health and developmental outcomes among populations facing risks by increasing parent support for learning and development, promoting parent well-being, and preventing child maltreatment (Pew Home Visiting Project 2015). The ongoing Home Visiting Evidence of Effectiveness (HomVEE) review, commissioned to identify evidence-based programs and guide agencies implementing Maternal, Infant, and Early Childhood Home Visiting (MIECHV) programs, reflects this overall approach (Sama-Miller et al. 2017). Specifically, 20 home visiting models with demonstrated success addressing one or more of eight domains (child health; child development and school readiness; family economic self-sufficiency; linkages and referrals; maternal health; positive parenting; reductions in child maltreatment; and reductions in juvenile delinquency, family violence, and crime) have been designated as evidence-based. The current study was undertaken to look inside individual home visits, all conducted following guidelines from a model program and funded under the auspices of MIECHV, to examine active intervention ingredients used and their relations with ratings of home visit quality.

### Home Visiting Programs—Purposes and Theory of Change

Model home visiting programs focus on working in the family’s home and target relationships among the child’s immediate family members based on both theoretical and empirical support. Ecobehavioral theory (Bronfenbrenner 1961, 1994) posits that child development is influenced most strongly by daily environments (e.g., interactions within the immediate family and home) and further, that supportive relationships with extended family members, a vibrant community, and family-friendly government policies can influence overall family functioning and child development positively, and has influenced practice, policy, and research over the past half century. As well, empirical knowledge about early development is expanding rapidly. Responsive and stimulating care promotes optimal child development (Bornstein and Tamis-LeMonda 1989), even in the face of risks (Egeland et al. 1993; Werner 2000), and is especially important early in life when it sets patterns for secure attachment, sensory pathways, language, and cognitive functioning (National Scientific Council on the Developing Child 2007) that will influence the individual throughout life (Collins et al. 2000).

Evidence that responsive caregiving promotes healthy development is reflected in widely-used home visiting models. The majority (15 of 20) of evidence-based home visiting models (Sama-Miller et al. 2017) target caregiving directly; this reflects an underlying theory of change that enhancing parents’^1^ ability to provide responsive and developmentally supportive care is the active ingredient enabling home visiting programs to effect positive outcomes. Home visiting programs, however, often do not have an explicitly stated theory of change to describe specific mechanisms used to achieve intended outcomes (Weiss 1995).

### Home Visiting Process, Content, Quality, and Outcomes

Practice recommendations (Roggman et al. 2008a) also reflect this theory of change by encouraging active engagement of parents with their children during home visits. Triadic interactions that involve the child, parent, and home visitor working collaboratively promote engagement of all participants and focus content on enhancing parent–child interaction and child development (Hughes 2005; McCollum and Yates 1994). This sets the stage for quality practices and reflects program goals across home visiting models and services.

Triadic interactions, unfortunately, occur for small proportions of time during many home visits. Triadic interactions were observed 40 and 27% of the time during Part C and Early Head Start (EHS) home visits respectively (Peterson et al. 2007). Triadic interactions provide opportunities for home visitors to observe parents’ interactions with their child, but what home visitors say and do to support these interactions is key. The home visitor can model specific types of interaction strategies the parent might use when that is necessary. Likely more helpful, the home visitor can coach the parent providing specific suggestions for activities to do, specific language to use with their child and cues regarding the child’s communicative signals along with ways to respond to their child’s cues.

Coaching parent–child interactions can strengthen parents’ competence for promoting their child’s development as well as boost parental confidence and enjoyment of the child. Home visits, in an EHS program, were rated as higher quality when facilitating parent–child interaction was a goal (Roggman et al. 2001). In contrast to parent education approaches that emphasize sharing developmental information by talking with parents, active coaching gives opportunities for parents to practice new activities and ways of interacting with their young children while the home visitor provides encouragement and feedback. It also affords opportunities for home visitors to identify and support parent’s recognition of and differential response to their child’s cues, which is linked to sensitive and responsive caregiving (Roggman et al. 2008a).

Family engagement and a focus on child development, which are promoted with triadic interactions, were associated with more positive outcomes in EHS home visiting programs (Peterson et al. 2013; Raikes et al. 2006), perhaps in part because these same things were associated with longer duration of program enrollment (Roggman et al. 2008b). Enhanced caregiving outcomes resulting from home visiting programs have been linked to positive child development outcomes (Olds et al. 2002, 2004) with later child outcomes attributed directly to earlier caregiving outcomes among families participating in EHS home visiting programs (Raikes et al. 2014). In general, strength-based practices have been shown to promote a more supportive home environment for the child (Green et al. 2004).

### Research Questions

In the current study, direct observation, was used to relate specific home visiting strategies with quality ratings of home visits. Specific questions guiding this research included:

1. What proportion of home visit time was spent in triadic interactions?
2. What was the nature of the home visitor’s activity (i.e., observing, modeling, coaching) during triadic interactions?
3. What were the unique contributions of observing, modeling and coaching to ratings of home visit quality?

## Method

### Iowa MIECHV Programs

Iowa MIECHV programs were initiated in ten communities across the state; preliminary work by the Iowa Department of Public Health (IDPH) identified participating communities as those with the highest proportions of families facing multiple risks (e.g., premature birth, child maltreatment, poverty, substance abuse, domestic violence). These 10 sites, across 18 counties, are home to families living in both rural and urban communities.

The IDPH contracts with local agencies to deliver MIECHV services which were initiated in 2014. Agency applicants were asked to identify one of four models for implementation: EHS, Healthy Families America (HFA), Nurse Family Partnership (NFP), or Parents as Teachers (PAT). These four models are the most widely used evidence based models funded by MIECHV. In addition, each of these models targets the parent–child relationship as a primary mechanism for enhancing child development. The current study was embedded in a larger evaluation of Iowa MIECHV programs. Participation in the evaluation was voluntary for families; home visitors were required to participate in some evaluation activities based on employment status, but home visitors voluntarily consented to have data concerning their demographic information included in research examinations presented here. All activities were approved by the university’s institutional review board.

### Participants

Participants included families who received MIECHV-funded home visiting services and their home visitors. Table 1 presents demographic information for families (1) served by MIECHV-funded programs, (2) who consented to be a part of the evaluation study, and (3) included in the final analysis sample. Groupings were not mutually exclusive; the evaluation sample and analysis sample were subsamples of MIECHV enrollees, and the analysis sample was a subsample of the evaluation sample. One-way ANOVA was used to examine primary caregiver age, household size and annual income revealing no statistically significant differences among these groups. Chi square was used to compare samples on marital status (married vs. never married), education, and race of primary caregiver (White vs. non-White). One difference was found among groups; the analysis sample included a greater proportion of caregivers who reported having at least some college than the overall MIECHV sample [χ^2^(4) = 19.78, *p* < .001]. One hundred and eight home visits, one per family, were observed (84 HFA, 10 EHS, and 14 NFP).

**Table 1:** Demographic characteristics by sample (primary caregiver data)

	MIECHV sample (*N* = 1886)		Evaluation sample (*n* = 440)		Analysis sample (*n* = 108)	
	*M* (*SD*)	Percentage	*M* (*SD*)	Percentage	*M* (*SD*)	Percentage
Age^a^	24.24 (6.83)		23.80 (5.68)		24.07 (5.84)	
Household income	$12,897 (13,553)		$13,957 (14,094)		$15,373 (14,936)	
Household size	3.25 (1.50)		3.12 (1.47)		3.22 (1.49)	
Gender (% female)		99		99		99
Education^b^						
Less than high school		22		17		13
Diploma/GED/enrolled in HS		48		49		44
At least some college		28		34		43
Ethnicity (% Hispanic)		14		15		21
Race^c^						
White		60		62		65
Black or African American		20		18		11
Asian/Pacific/Native		3		2		0
Multiracial		17		18		24
Marital status^d^						
Never married		74		75		69
Divorced		4		5		7
Separated		2		2		3
Married		19		18		21
Child gender (% female)^e^		48		49		47

Where percentages do not equal 100% there is missing data or rounding. For statistical tests, categories were collapsed for caregiver race and marital status due to small numbers in sub-categories

^a^4 = missing in MIECHV sample

^b^2 = missing in Evaluation sample, 28 = missing in MIECHV sample

^c^1 = missing in Analysis sample, 1 = missing in Evaluation sample, 4 = missing in MIECHV sample

^d^1 = missing in Evaluation sample, 6 = missing in MIECHV sample

^e^14 = missing in Evaluation sample, 255 = missing in MIECHV sample

Home visitors implemented home visiting models [HFA (*n* = 33), EHS (*n* = 7) or NFP (*n* = 5) models] selected by their employing agencies. All 45 home visitors were female, 89% identified as White, and 75% were younger than 40. The majority spoke only English (87%) and had at least a bachelor’s degree (89%). Their average length of employment as a home visitor was 3.5 years (*SD* = 7.5); most home visitors had been in the field about 1 year. Number of observations per home visitor ranged from 1 to 7 (*M* = 2.40, *SD* = 1.81); more than half of home visitors (21) contributed a single video; ten contributed two videos; three contributed three, four, and five respectively; four contributed six videos; and one home visitor contributed seven videos.

### Measures

Family demographic information was collected by home visitors at program enrollment. Home visitors self-reported information about their personal characteristics, education and training. Home visitors were asked to send a video-recording of one home visit with each family annually; a staff member from the home visiting program (e.g., the home visit supervisor) recorded the visits.

#### Observations

Trained research assistants used the Home Visit Observation Form-Revised (HVOF-R: McBride and Peterson 1996) and the Home Visit Rating Scales-Adapted & Extended to Excellence (HOVRS A+: Roggman et al. 2012) to assess home visit characteristics and quality. The HVOF-R facilitates simultaneous coding of data in three broad categories, with each category further divided into mutually exclusive subcategories: (1) primary interaction partners (e.g., parent–home visitor, parent–child, home visitor–child, joint home visitor–parent–child), (2) content of interaction (e.g., child’s development, parenting issues), and (3) nature of the interventionist’s interaction (e.g., observing, modeling, coaching, providing information, paperwork). Each category is coded simultaneously during 30-s observation intervals allowing for description of who was interacting with whom, the content of the interaction, and the home visitor’s specific role in the interaction during each interval (see Peterson et al. 2007 for complete description of the HVOF-R).

For the current study, items were selected, conceptually, to represent triadic interactions. Intervals were identified as triadic interactions if primary interaction partners was coded as joint home visitor–parent–child and nature of the home interventionist’s interaction was coded as observing, modeling or coaching. Overall percentages of time spent in triadic interactions were calculated for each home visit.

Each observer established inter-observer agreement at or > 85% overall with no single category < 80% on three consecutive observations before beginning independent data collection. Team members met weekly to discuss disagreements and code, by consensus, to ensure reliability of the data. The detailed nature of the HVOF-R interval coding system makes training observers time-consuming; consensus coding was used to train new observers, re-train observers if an observation fell below expected levels of agreement, and to clarify code definitions when necessary. Consensus coding was used for 56 observations, and the remaining 52 observations were coded independently with 16 coded by two independent observers. Across all categories, average interrater agreement was 89% with rates for each category as follows: primary interaction partners (94%), interaction content (88%), and nature of the interventionist’s interaction (85%).

The HOVRS A+ facilitates quality ratings of home visit practices and engagement. Scores from four domains (home visitor responsiveness to family, home visitor relationship with family, home visitor facilitation of parent–child interaction, and home visitor non-intrusive collaboration) are averaged to provide a quality rating for home visit practices which captures the ways home visitors facilitate meaningful interactions between caregivers and children that promote developmental parenting behaviors. Scores from three domains (parent–child interaction, parent engagement, and child engagement) are averaged to provide a quality rating of overall engagement in home visit activities. An observer watches an entire home visit and then rates each of the seven domains from 1 (poor quality) to 7 (excellent quality) based on presence of a continuum of domain-specific behavioral indicators.

Interrater agreement, based on developer criteria of 85% of items within one point within each domain, across three consecutive observations was achieved before an observer coded independently. Every fourth observation was coded by two observers to monitor interrater agreement. Average interrater agreement rates for each domain were: home visitor responsiveness to family (100%), home visitor relationship with family (97%), home visitor facilitation of parent–child interaction (100%), home visitor non-intrusive collaboration (90%), parent–child interaction (100%), parent engagement (100%), and child engagement (100%).

### Data Analyses

Data captured with the HVOF-R were used to answer the first two research questions. Data from all 108 home visits were merged to calculate the overall proportion of home visit time spent in triadic interactions, as well as the proportions of time during triadic interactions that home visitors spent observing, modeling and coaching.

For the third research question, proportions of time home visitors spent in these specific activities were examined in relation to home visit quality as measured by the HOVRS A+. Multilevel regression modeling controlled for the nesting of individual family home visits among the home visitors. Length of home visit was included in order to control for opportunity (time) home visitors had to engage in observation, modeling and coaching.

## Results

Preliminary analyses revealed that home visit length ranged from 20 to 90 min (*M* = 46, *SD* = 16.04). Substantive results are presented below.

### Time Spent in Triadic Interactions and Home Visitors’ Strategies

**Fig. 1:**
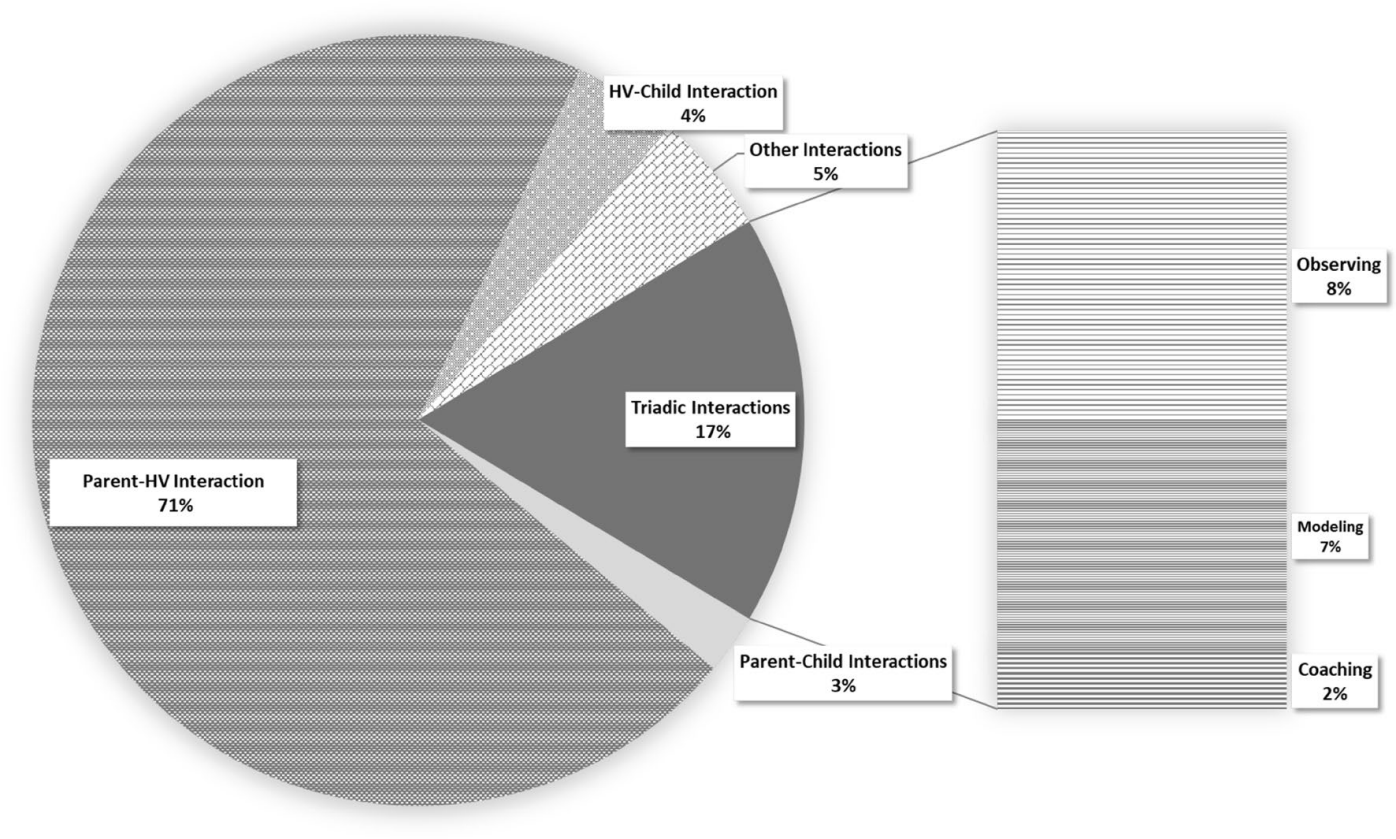
Proportion of home visit time by interaction partners and home visitor activity

First, interaction patterns were examined across all home visits (see Fig. 1). Home visitors spent almost three-quarters of their time interacting with the parent/s, with only 17% of the time spent in triadic interactions, the focus of this study. Given a mean home visit length of 46 min, on average, < 10 min of each visit was devoted to triadic interactions.

Examination of home visitors’ activities during triadic interactions revealed only 2% of each visit, on average, was spent coaching parent–child interactions. The remaining 15% of time spent in triadic interactions was divided evenly between home visitors observing parent–child interactions (8%) and modeling interaction for the parent (7%).

### Relating Home Visit Activities to Quality Ratings

Next, we examined how home visitors’ time spent in triadic interactions was related to home visit quality. Table 2 presents data to describe home visit activities and quality, as well as correlations among home visit length; proportion of triadic interaction time the home visitor spent observing, modeling and coaching (from the HVOF-R); and quality ratings of home visit practices and engagement (from the HOVRS A+). Length of home visit was related only to home visit practices quality. Proportions of time the home visitor spent observing, modeling and coaching during triadic interactions were related to quality ratings of both home visit practices and engagement with one exception—observing was not related to the quality of home visit practies.

**Table 2:** Mean, standard deviations, and intercorrelations for quality scores and home visit interactions (N = 108)

Measure	*M*	*SD*	1	2	3	4	5	6	7
1. HV length	46.14	16.04	–						
2. % Triadic	17.31	13.07	.12	–					
3. % Observing	8.62	9.91	.12	.64**	–				
4. % Modeling	6.93	7.15	.13	.68**	.05	–			
5. % Coaching	1.67	2.50	.14	.44**	− .01	.29**	–		
6. HV practices quality	3.21	.74	.28**	.33**	.04	.23*	.36**	–	
7. Engagement quality	4.06	.94	.11	.51**	.28**	.34**	.31**	.60**	–

*p < .05; **p < .01

To examine the unique contributions of observing, modeling and coaching to prediction of quality ratings of home visit practices and engagement, data were submitted to multilevel regression modeling analyses. This strategy was selected because approximately half the home visitors conducted visits with multiple families, and the interclass correlations indicated a nesting effect within home visitor for quality ratings of home visit practices (*r* = .30) and engagement (*r* = .13). Therefore, Level 1 variables included: (1) length of visit (control) and percentage of time the home visitor spent (2) observing, (3) modeling, and (4) coaching. Home visitor was included as a Level 2 variable.

The proportion of time home visitors spent coaching predicted quality ratings of home visit practices after accounting for length of visit and proportions of time spent observing and modeling as presented on Table 3. Proportions of time spent observing and modeling did not predict quality ratings of home visit practices. Time spent observing, modeling and coaching each uniquely predicted quality ratings of engagement.

**Table 3:** Multilevel model predicting quality of home visit practices and engagement (N_visit_ = 108, N_homevisitor_ = 45)

	HOVRS A+ quality of home visit practices				HOVRS A+ quality of engagement			
	*b*	*p*	*SE*	95% CI	*b*	*p*	*SE*	95% CI
Observing	.00	ns	.01		.03	***	.01	
Modeling	.01	ns	.01		.03	**	.01	
Coaching	.08	**	.03		.09	**	.03	
Length of visit	.01	*	.00		.00	ns	.01	
Random effects								
Residual variance	.41		.08	.28–.61	.60		.11	.42–.85
Home visitor random effects	.03		.07	− .00 to 3.80	.07		.09	.01–.82

*p ≤ .05; **p ≤ .01; *p* ≤ .001

## Discussion

The theory of change guiding most home visiting programs that target families with risks, identifies the parent–child relationship and interactions as the primary mechanism for improving child development outcomes. Enhancing parent–child interactions is the stated focus of multiple model programs (e.g., EHS, HFA, PAT), and researchers have demonstrated that improving these interactions is related positively to later child development outcomes (Raikes et al. 2014). Parent engagement and a focus on child development content, important quality indicators for home visits (Korfmacher et al. 2008; Roggman et al. 2001), are related to more positive child and family outcomes in the short term (Raikes et al. 2006), as well as over time (Peterson et al. 2012).

We examined use of triadic interactions, captured with the HVOF, to identify specific strategies, namely observing interactions, modeling for the parent and coaching the parent during parent–child interactions, home visitors use that relate positively to quality ratings of home visit practices and engagement captured with the HOVRS-A+. Very little time during the observed home visits, unfortunately, included simultaneously engaging the caregiver and child in home visit activities, similar to findings from earlier research (Peterson et al. 2007). The majority (71%) of home visit time spent in home visitor–parent interactions suggests a priority on engaging parents in discussions of child and family issues rather than supporting parent–child interactions directly. Even more striking is that of the average 17% of home visit time spent in triadic interactions, the vast majority of that time (15%) was spent either observing interactions or modeling for the parent rather than directly coaching parent–child interactions.

Data from the current observations document that all triadic intervention strategies home visitors used (observing, modeling, and coaching) engage parents and children as evidenced by their relation to quality ratings of engagement. However, coaching parent–child interactions predicts quality ratings of home visit practices even after controlling for time spent observing and modeling. Coaching facilitates parent–child interactions and promotes collaboration when parents and home visitors work together to identify play activities and/or daily routines they would like to improve. While coaching, home visitors provide the context for a variety of high quality practices that enhance parent–child interactions targeted by most home visiting programs. Coaching parent–child interactions was associated with higher rates of maternal engagement during home visits, especially among parents facing significant risks (e.g., teenagers, low levels of education; Peterson et al. 2007). Despite low frequency, the significant relationship between coaching and quality ratings of home visit practices suggests that active coaching may be a particularly powerful mechanism for enhancing overall home visit quality and ultimately child outcomes.

It is important to note that home visit length was related to quality ratings of home visit practices but not quality ratings of engagement. Bivariate correlations revealed that neither engaging in triadic interactions or family engagement is related systematically to length of visit alone. Together, this suggests that asking home visitors to devote significant portions of each visit to building rapport in order to engage family members is unlikely to be an effective strategy. As well, triadic interactions appear to be the necessary but not sufficient floor for engaging parents in meaningful interactions with their children. Facilitating effective parent–child interactions that can provide opportunities for home visitors to build parents’ competence through coaching takes not only time but sophisticated skills that allow home visitors to recognize interaction opportunities and capitalize on them. This unique relationship between coaching activity and quality ratings of home visit practices presents several important implications for professional development, but study limitations will be discussed first.

### Limitations

The current study used data about individual home visits from a subset of families who participated in evaluation of Iowa’s MIECHV programs. While there were few differences between families participating in the evaluation and all families enrolled in the home visiting programs, the sample was limited in size and only includes home visits conducted in English. It is possible that home visits observed for this study may not be representative of the home visitors’ overall practices or all families’ experiences.

Only one visit per family was observed. Ongoing examination of the HVOF-R has demonstrated that observing for 40–60 min provides a stable estimate of overall home visit activities for an individual family (Peterson et al., under review). Limited and inconsistent numbers of observations by home visitors made it impractical to examine practice differences within home visitor (across families). Finally, the majority of visits observed were from the HFA home visit model. This is representative of overall MIECHV services across the state, and the HFA model is used widely across the country. However, further examination of similarities and differences in home visitors’ activities across evidence-based models is needed for a more thorough understanding of the most important intervention elements of each model.

### Implications

The primary practice implication of the current study is the need to increase the proportion of home visit time spent in triadic interactions. Relations between use of triadic interactions and quality ratings presented here align with other evidence emerging to support this practice. First, HOVRS-A+ quality ratings, which are higher when greater proportions of home visit time are devoted to triadic interactions, predict positive outcomes for both parents and children participating in home visiting programs (Roggman et al., under review). Positive communication outcomes among toddlers with disabilities resulted from interventionists coaching interactions while engaging the parent and child in triadic interactions (Brown and Woods 2015).

While positive relations between use of triadic interaction strategies and ratings of effectiveness quality is not surprising given the operational definitions used in each code, data presented here help identify interaction patterns and specific behaviors home visitors can implement to maximize program efficacy. Triadic interactions set the stage for enhancing parent–child interactions, strengthening parent–child relationships, and building parenting efficacy. Families are very diverse and strengthening the skills of multiple caregivers to interact effectively with young children may be important in many families. For example, purposeful inclusion of father figures in home visit activities may be particularly effective in promoting child development outcomes across domains (Cabrera et al. 2007). Rowe (2018) found father figures present in the home during approximately 25% of visits. When fathers were present, they were available for interaction over 75% of the time, on average, but were included by the home visitor only 43% of the time indicating a need to provide home visitors further professional development in this area.

Increasing the amount of time spent in triadic interactions will necessarily mean decreasing the amount of time spent in other types of interactions, especially time spent in parent–home visitor interactions. Parent–home visitor interactions are necessary for sharing information about the program, as well as information about community resources and educational opportunities. Likely, some parent–home visitor interaction time is needed to ensure open communication and a trusting relationship, but if optimizing child outcomes and enhancing parent–child relationships continue to be a primary target for home visiting programs, home visitor–parent interactions should not be to the exclusion of coaching parent–child interactions directly. In short, home visits need to reflect more action and less talking, but this is much easier to say than do.

Many home visitors will need support to increase use of triadic interactions, and more specifically coaching, which should be a central focus of professional development for home visitors and continuous improvement activities for programs. Effective home visiting involves a complex set of interactions and contexts. Likely, short-term focus on one specific behavior will not be effective; rather attention to knowledge, skills, and attitudes home visitors need to build relationships with families facing risks while helping them build competencies will be needed (Roggman et al. 2016). Home visitors must have knowledge not only about child development, but also about family systems, families’ beliefs and cultural practices, and challenges faced by families living in poverty. Home visitors need skills to engage families in relationships focused on supporting their children, help parents build knowledge and skills, and enhance parent–child interactions. Finally, home visitors need attitudes that recognize parents’ expertise on their own children, accept flexibility, and promote self-reflection. Roggman and colleagues (2016) provide recommendations for higher education providers, but some of the same recommendations could guide professional development efforts for current home visitors and their agency colleagues.

Recently, programs have begun to experiment with virtual home visiting. Virtual home visits depend on a unique form of triadic interactions; while the home visitor is not in physical proximity to the parent and child, joint interaction among the participants is key to engagement and communication. Home visitors actually coach parent–child interactions more during virtual home visits as compared to in-person visits; likely, the home visitor’s lack of direct physical contact with the child necessitates working through the parent to engage the child, the essence of coaching parent–child interactions (Behl et al. 2017). Lessons learned from these programs may give valuable information about how to train and support both home visitors and their supervisors around increasing the use of coaching strategies.

Close examination of overall agency functioning, how supervisors work with home visitors, and how program administrators arrange training and support is needed as well. High quality infrastructure supports for home visiting programs is related to higher quality home visits (Korfmacher et al., under review) and is evidenced by more thorough supports for direct service providers. For example, direct observations, either in real time or through video recording, can provide essential information for reflective supervision that can maximize the valuable time resources of home visitation. Decreasing the amount of home visit time devoted to paperwork and other administrative tasks may also be fruitful. This may call for examining overall reporting requirements, as well as efficiency for meeting those requirements.

Home visiting programs are administered by a variety of social services, health, and education agencies, all of which have demonstrated efficacy in a variety of locations. Fortunately, most home visiting programs serving families with young children share relationship-based collaborative elements that can be observed, measured and supported to help guide professional development efforts. While no specific “administrative home” may be necessary to ensure program effectiveness, attention to supervisory and professional development supports necessary to achieve and sustain high quality services will be key and may require oversight from local, state, and federal policy makers and funders.

## Footnotes

1 We acknowledge that families differ, and children may have caregivers other than their biological parents (e.g., grandparent, aunt, older sibling). In this paper, we use the generic term, parent to represent the adult/s who take primary responsibility for daily caregiving and decision-making for the child/ren in the family.
