# Correction to: Triadic Interactions in MIECHV: Relations to Home Visit Quality

**DOI:** 10.1007/s10995-018-2620-0

**Published:** 2018-08-22

**Authors:** Carla A. Peterson, Kere Hughes-Belding, Neil Rowe, Liuran Fan, Melissa Walter, Leslie Dooley, Wen Wang, Chloe Steffensmeier

**Affiliations:** 1 Human Development and Family Studies, Iowa State University, 4380 Palmer, Ames, IA 50011 USA; 2 Ames, USA

## Correction to: Maternal and Child Health Journal 10.1007/s10995-018-2534-x

The article “Triadic interactions in MIECHV: Relations to home visit quality”, written by Carla A. Peterson, Kere Hughes-Belding, Neil Rowe, Liuran Fan, Melissa Walter, Leslie Dooley, Wen Wang and Chloe Steffensmeier, was originally published electronically on the publisher’s internet portal (currently SpringerLink) on 12 June 2018 without open access. With the author(s)’ decision to opt for Open Choice the copyright of the article changed on 9 July 2018 to © The Author(s) 2018 and the article is forthwith distributed under the terms of the Creative Commons Attribution 4.0 International License (http://creativecommons.org/licenses/by/4.0/), which permits use, duplication, adaptation, distribution and reproduction in any medium or format, as long as you give appropriate credit to the original author(s) and the source, provide a link to the Creative Commons license and indicate if changes were made.

The original article has been corrected.

